# Genetic Variation in the Human Brain Dopamine System Influences Motor Learning and Its Modulation by L-Dopa

**DOI:** 10.1371/journal.pone.0061197

**Published:** 2013-04-17

**Authors:** Kristin M. Pearson-Fuhrhop, Brian Minton, Daniel Acevedo, Babak Shahbaba, Steven C. Cramer

**Affiliations:** 1 Department of Anatomy & Neurobiology, University of California Irvine, Irvine, California, United States of America; 2 Department of Statistics, University of California Irvine, Irvine, California, United States of America; 3 Department of Neurology, University of California Irvine, Irvine, California, United States of America; INSERM/CNRS, France

## Abstract

Dopamine is important to learning and plasticity. Dopaminergic drugs are the focus of many therapies targeting the motor system, where high inter-individual differences in response are common. The current study examined the hypothesis that genetic variation in the dopamine system is associated with significant differences in motor learning, brain plasticity, and the effects of the dopamine precursor L-Dopa. Skilled motor learning and motor cortex plasticity were assessed using a randomized, double-blind, placebo-controlled, crossover design in 50 healthy adults during two study weeks, one with placebo and one with L-Dopa. The influence of five polymorphisms with established effects on dopamine neurotransmission was summed using a gene score, with higher scores corresponding to higher dopaminergic neurotransmission. Secondary hypotheses examined each polymorphism individually. While training on placebo, higher gene scores were associated with greater motor learning (p = .03). The effect of L-Dopa on learning varied with the gene score (gene score*drug interaction, p = .008): participants with lower gene scores, and thus lower endogenous dopaminergic neurotransmission, showed the largest learning improvement with L-Dopa relative to placebo (p<.0001), while L-Dopa had a detrimental effect in participants with higher gene scores (p = .01). Motor cortex plasticity, assessed via transcranial magnetic stimulation (TMS), also showed a gene score*drug interaction (p = .02). Individually, DRD2/ANKK1 genotype was significantly associated with motor learning (p = .02) and its modulation by L-Dopa (p<.0001), but not with any TMS measures. However, none of the individual polymorphisms explained the full constellation of findings associated with the gene score. These results suggest that genetic variation in the dopamine system influences learning and its modulation by L-Dopa. A polygene score explains differences in L-Dopa effects on learning and plasticity most robustly, thus identifying distinct biological phenotypes with respect to L-Dopa effects on learning and plasticity. These findings may have clinical applications in post-stroke rehabilitation or the treatment of Parkinson's disease.

## Introduction

Dopamine is a neurotransmitter that has a key role in numerous brain processes including movement, reward, learning, and plasticity [1]. Polymorphisms in the genes encoding for dopamine receptors and degradation enzymes contribute to inter-individual differences in some forms of learning [2], with polymorphisms that reduce dopamine neurotransmission thought to impair learning and cognitive performance, and those that increase dopamine neurotransmission improving these behaviors [3], [4]. These genetic influences on dopamine-related learning are thought to be paralleled by gene effects on brain plasticity [5]; for example, studies of cognitive flexibility and working memory show differences in prefrontal and striatal activation in relation to variation in dopamine genetics [6], [7], [8]. However, questions remain whether these genetic influences extend to the brain motor system and to dopaminergic therapies that target this system.

Several prior results suggest the possibility that variation in the genetics of dopamine neurotransmission might affect motor learning and motor cortex plasticity. Abundant evidence supports a role of dopamine in learning and cortical plasticity in the motor system [9], [10], [11]. Furthermore, the principle that genetic variability can influence motor learning and motor cortex plasticity in humans has been established, mainly with the val^66^met polymorphism in the gene for brain derived neurotrophic factor (BDNF) [12], [13]. However, little is known regarding the influence that dopamine-related gene variants have on learning and cortical plasticity in the healthy motor system. This is also true in the clinical pharmacological setting, where inter-individual differences are common, and genetic variation might be a significant factor. For example, variability in response to dopaminergic therapy for Parkinson’s disease is high [14]. Another example is drugs to promote brain plasticity after neural injury such as stroke, where results to date have been inconsistent [15], [16], [17], with motor learning and plasticity improved by dopaminergic drugs in some studies [18], [19] but not in others [20], [21], [22]. Together, these findings suggest that variation in dopamine genetics might be useful to understand individual differences in motor system function in healthy and in clinical therapeutic settings.

A key challenge to understanding the influence of genetic variation is that a large number of proteins affect dopamine neurotransmission. This issue was addressed in the current study by examining the collective effect of multiple polymorphisms. Five polymorphisms known to influence brain dopamine neurotransmission were chosen for the current investigation, with their combined effect examined as a gene score, an approach that has been found useful for calculating genetic risk in several human disease settings [23], [24], [25]. The five genes of interest are catechol-o-methyltransferase (COMT) and the dopamine transporter protein (DAT), which regulate synaptic dopamine levels, along with dopamine receptors D1, D2 and D3. These five proteins are widely distributed throughout the brain: DRD1 and DRD2 are widespread throughout the brain, found in both cortex and basal ganglia [26], [27], [28], [29], [30], [31], [32], [33], DRD3 is found in the ventral striatum [33], [34], [35], DAT protein is found primarily in the basal ganglia [29], [31], [36], [37], [38], and COMT is found in both striatum and cortex [39]. Together these proteins subserve dopamine neurotransmission across motor cortices, basal ganglia, and other brain structures relevant to motor learning [40], [41], [42], [43]. The effect of genetic variation in these five proteins was examined collectively, via a gene score, as well as separately for each polymorphism.

The current study examined the impact that genetic variation in the dopamine system has on skilled motor learning, motor cortex plasticity, and their modulation with L-Dopa. Using a randomized, double-blind, placebo-controlled crossover design, the three main study hypotheses were (1) genetic polymorphisms affecting dopaminergic neurotransmission influence skilled motor learning and motor cortex plasticity in the normal state, i.e., on placebo, (2) elevating brain dopamine levels via L-Dopa administration improves learning and plasticity, and (3) the influence of L-Dopa varies in relation to dopamine genetics. The effects of these genetic polymorphisms were examined collectively as well as separately, in order to determine if genetic associations with a polygene score are stronger than with any individual gene.

## Materials and Methods

### Participants

Participants were recruited from the University of California, Irvine campus and surrounding areas. Entry criteria were 18–35 years old, right-handed, no neurological or psychiatric conditions, and not using prescription or recreational dopamine-enhancing drugs. In order to focus on dopamine genetics, participants with the BDNF val^66^met polymorphism, which is known to have a significant effect on motor learning and motor cortex plasticity [12], [13], [44], [45], were excluded, and so only individuals whose BDNF genotype was val/val were included in the study.

#### Ethics statement

Participants provided written informed consent. The study was approved by the Institutional Review Board of the University of California, Irvine.

### Protocol

**Figure 1:**
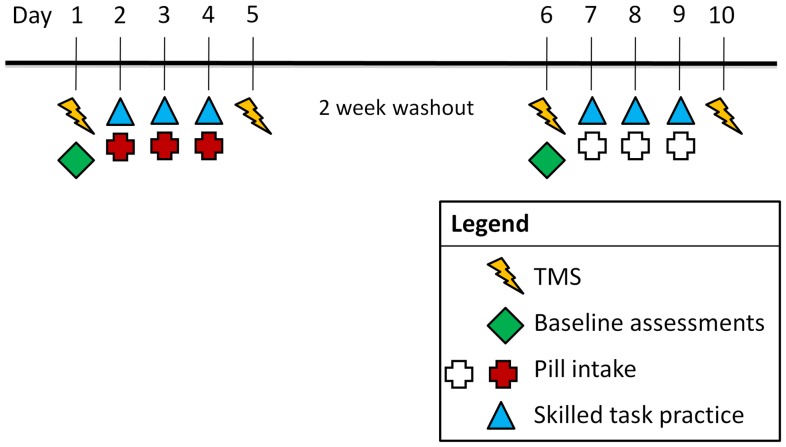
The experimental protocol.

**Figure 2:**
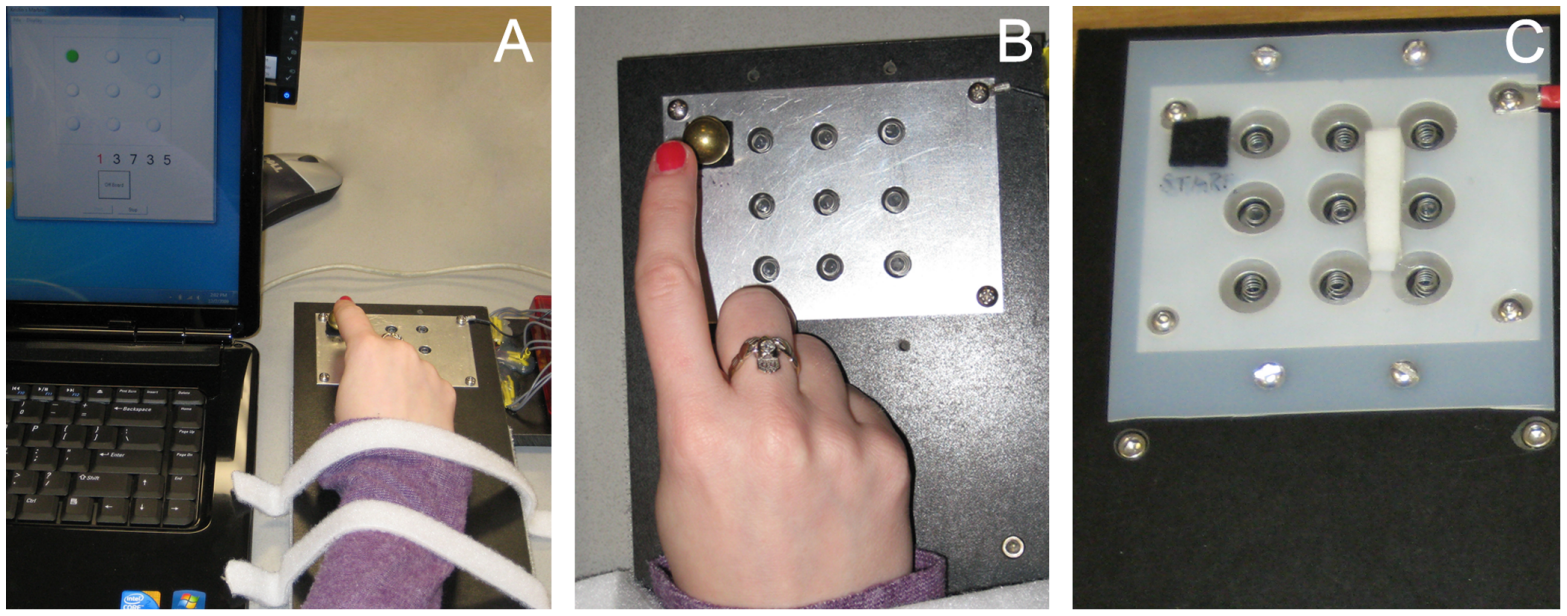
The Marble Navigation Task used to measure skilled motor learning. (A) Participants view the computer screen to see where to move the marble. (B) The MNT board used for week 1. (C) A novel version of the same MNT board was used for week 2.

The genotype for each participant was determined for five dopamine-related polymorphisms. Participants were then randomized to a pill group (L-Dopa followed by placebo vs. placebo followed by L-Dopa) in a double-blind manner. For each of the two weeks, the protocol was five days in duration (Figure 1). On day 1 of each week, transcranial magnetic stimulation (TMS) of the left hemisphere was used to measure the pre-training area of representation for the right first dorsal interosseus (FDI) muscle, following a published protocol [46]. Following TMS, a baseline measure of performance on the marble navigation task (MNT, Figure 2A), a skilled motor task that places intensive demands on the FDI muscle, was obtained over 100 targets. On day 2, participants took the first pill of the week, waited for 1 hour, and then performed four 100-target MNT trials (400 targets total). This pill+training was repeated two more days, for a total of three days, a time interval sufficient for L-Dopa effects to emerge [47]. On day 5, one day after the week’s last pill, post-training TMS measures were obtained.

After a 2-week washout period, participants returned to repeat the protocol, with two differences: (a) participants took the pill (placebo vs. L-Dopa) that was not taken during week 1, and (b) a novel version of the MNT was used (Figure 2B). This period was intended to achieve a pharmacological (effect of the first drug) and TMS-based (effect of the first week’s training on motor maps) washout. Participants’ skill at the MNT was not expected to return to baseline over 2 weeks, and so a behavioral washout was not expected; therefore, a different version of the MNT task was used during week 2.

### Transcranial Magnetic Stimulation

The right FDI map area was determined as described previously [48], using a Magstim 200-2 stimulator and 70-mm figure-of-eight coil (Magstim; Whitland, UK). In sum, participants were seated upright, while surface electromyography (EMG) was recorded from the right FDI (gain = 10,000x, bandpass filters 30–1,000 Hz) with ground electrodes over the wrist and elbow. A T1-weighted high resolution volumetric anatomical magnetic resonance imaging (MRI) template was registered to each participant using Brainsight stereotactic software (Rogue Research; Montreal, QC). A 1-cm^2^ grid was superimposed on the cortical surface to guide stimulation and to facilitate map generation.

The site of lowest motor threshold (LMT), defined as the site that requires the smallest intensity to produce a motor evoked potential (MEP) ≥100 µV in at least 4/7 left hemisphere pulses, was determined to the nearest 1% of stimulator output, and the LMT noted, separately for each of the four TMS sessions. Stimulation was then applied systematically at 110% LMT in 1-cm increments across the cortical surface in a spiral pattern surrounding the site of LMT. Positive sites, defined as sites that produced an MEP≥100 µV in at least 4/7 pulses, were noted. The mapping procedure was repeated until all positive sites were surrounded by negative sites that did not produce this response, thereby generating the cortical motor map for right FDI.

### Marble Navigation Task

The MNT required individuals to navigate a marble through a series of target wells using their right index finger, a task chosen because it makes intense and skillful demands on the right FDI muscle (Figure 2), and has been shown to produce M1 plasticity over several days [49]. Participants were seated in front of a board that had 9 shallow (1 mm) target wells arranged in a 3×3 grid. The wrist and forearm were fixed to the board with Velcro straps to maximize reliance on the FDI, the index finger was held in a semi-flexed position over the grid, and a 14 mm diameter metal marble was placed on a felt starting square. A computer screen displayed one of the 9 wells as the target. The participant moving the marble into a well triggered an electrical connection. If the well was the correct target, a tone sounded and the computer displayed the next target well; if the well was not the target, a lower-pitch tone sounded and the computer recorded the error. Each trial was composed of 100 self-paced targets; the computer did not display the next target until the current target was reached. Time to completion (TTC) of the task, measured as the time between the first marble contact and correct completion of the 100^th^ well, was recorded. Four 100-well trials were performed on each of the three training days (days 2–4 in week 1, and days 7–9 in week 2), providing approximately 20 minutes total of training per day. Subjects thus learned the motor movements necessary to manipulate the marble skillfully in response to a visual cue.

In pilot studies, improvements in TTC reached a plateau by one week of MNT training. In order to ensure that further motor skill learning occurred during the second study week, a more difficult MNT board was created by adding obstacles (Figure 2B); pilot studies confirmed that skill learning continued (i.e., improvements in TTC were seen) on this second board after a plateau had been reached on the first board. The order of these two tasks was therefore not randomized, with all subjects using the obstacle-free board during the first week and the more difficult board during the second week, in order to continue skilled learning during the second week.

### Pill Intake

Participants took a pill (after 2 hours fasting) before training for three days in each of the two weeks (Figure 1). At the start of these visits, participants took either L-Dopa (as part of Sinemet ® 25/100) or placebo (methylcellulose), one hour prior to MNT training, a timing interval that was selected to have MNT training coincide with peak L-Dopa blood levels [50]. Prior studies have found this dose to be effective in enhancing learning and plasticity [47], [51]. L-Dopa was selected as the method to increase brain dopamine because it is predominately metabolized to dopamine, less than 5% of which is further converted to norepinephrine [52], [53], [54]. To maintain the double-blind, both L-Dopa and placebo were identically overencapsulated (Drug Product Services Laboratory, Dept. Clinical Pharmacy, UCSF). To standardize experience during the hour of pill absorption, participants were given non-motor activities such as watching video clips.

### Genotyping

DNA for genotyping was extracted from whole blood by salt precipitation. Genotyping for all polymorphisms was performed using polymerase chain reaction (PCR) - restriction fragment length polymorphism analysis. PCR products were digested with the appropriate restriction enzymes, digestion products were run on agarose gel and bands were visualized with ethidium bromide. Choice of primer sequences and digestion enzymes followed established protocols for COMT rs4680 [55], DAT rs28363170 [56], DRD1 rs4532 [57], DRD2/ANKK1 rs1800497 [58], DRD3 rs6280 [59] and BDNF rs6265 [60]. See Methods S1 for more details.

### The Dopamine Gene Score

A dopamine gene score was determined, representing the additive effects of five polymorphisms with established effects on dopaminergic neurotransmission (See Table 1). Initial analyses weighted all genes equally: single-gene genotypes that increase dopamine neurotransmission added +1 to the score and genotypes that decrease dopamine neurotransmission added 0 (see Table 1). Gene scores thus ranged from zero (lowest basal dopamine neurotransmission) to 5 (highest basal dopamine neurotransmission). Subsequent analyses examined a weighted gene score (see statistical analysis section).

**Table 1:** Summary of polymorphisms and classification for gene score.

	DRD1 rs4532			DRD2 rs1800497			DRD3 rs6280			COMT rs4680			DAT rs28363170		
	A/A	A/G	G/G	Glu/Glu	Glu/Lys	Lys/Lys	Ser/Ser	Ser/Gly	Gly/Gly	Val/Val	Val/Met	Met/Met	9/9	9/10	10/10
Classification	0	1	1	1	0	0	0	1	1	0	1	1	1	1	0
Predicted Frequency	0.47	0.49	0.04	0.48	0.4	0.14	0.5	0.35	0.15	0.37	0.49	0.15	0.06	0.33	0.56
Number in our sample	27	20	3	19	26	5	22	23	5	19	27	4	1	11	36
Frequency in our sample	0.54	0.40	0.06	0.38	0.52	0.10	0.44	0.460	0.10	0.38	0.54	0.08	0.02	0.22	0.72

The five polymorphisms related to brain dopamine neurotransmission are listed. Each was in Hardy-Weinberg equilibrium.

### Classification of Dopamine Variants

#### COMT (rs4680)

COMT is an enzyme that degrades catecholamines such as dopamine, and has a val^158^met polymorphism that results in a protein with 3–4 times lower enzymatic activity, and thus higher dopaminergic tone, based on multiple lines of evidence [61], including a positron emission tomography (PET) study finding that within several cortical areas, F-Dopa metabolism was greater in COMT val/val, compared to met/met, individuals [62].Presence of the val^158^met variant has been associated with greater working memory and prefrontal cortex physiology in human participants [3], [63]. The Val allele is associated with diminished performance on executive functioning and visuospatial tasks, and a decline in executive functioning with aging [64], [65]. Those lacking the Met variant show a greater response when amphetamine is added to enhance performance [7], [66]. Any presence of the val^158^met COMT variant increases dopamine neurotransmission (score = 1); val/val individuals were given score = 0.

#### DAT (rs28363170)

DAT is an enzyme that removes synaptic dopamine, and its gene has a 40 bp VNTR at the 3′ untranslated region that commonly occurs in either 9 or 10 repeats. The 9-repeat allele, as compared to the 10-repeat allele, is associated with less DAT protein and thus with greater synaptic dopamine neurotransmission [67], [68], [69]. Consistent with this, the 10-repeat allele has been associated with increased risk of attention deficit hyperactivity disorder (ADHD) [70], [71], [72], a hypodopaminergic state. Any presence of DAT 9 was given score = 1; and so participants with DAT 10/10, score = 0. There is evidence that uncommon 11-repeat alleles behave like 10-repeats, so all other DAT fragments were also coded as score = 0 [73].

#### DRD1 (rs4532)

DRD1 is a dopamine receptor, and while it is evolutionarily well-conserved and does not have any functional polymorphisms, its gene does have a −48 A/G SNP in the 5′ untranslated regulatory region [74]. Polymorphisms in such regulatory regions of genes can affect mRNA stability and translation, as described with DRD2 [75]. Consistent with this, the DRD1 G allele has been associated with several disorders associated with increased brain dopamine neurotransmission. For example, DRD1 disruption decreases nicotine self-administration in animals [76], [77], whereas the opposite effect is seen with the −48 G allele which is associated with an increased rate of nicotine dependence [78]. DRD1 and its −48 A/G SNP may also play a role in alcohol addiction [79]. In addition, the G allele is more common in persons with bipolar disorder [80], [81], and has also been implicated in traits such as compulsive eating, shopping, and gambling [82]–each of which is linked to increased brain dopaminergic tone [83]. The GG genotype is associated with an increased susceptibility for antipsychotic-induced tardive dyskinesia [84] along with treatment resistance in schizophrenia [85] and bipolar disorder [86]. For these reasons, presence of the G allele was classified as having increased dopamine neurotransmission (score = 1); A/A, score = 0.

#### DRD2 (rs1800497)

DRD2 is a dopamine receptor, and is associated with the ANKK1 TaqIA polymorphism, a Glu to Lys substitution at position 713. This is associated with a 40% reduction in the expression of D2 receptors in the striatum, as well as reduced D2 binding in post-mortem brain tissue, with Glu/Lys individuals showing significantly lower D2 binding than Glu/Glu homozygotes [87]. PET studies found reduced striatal D2 receptor availability with the Lys allele [88]. The Lys allele is also associated with predisposition to neuroleptic malignant syndrome, a hypodopaminergic state [89]. Further, the Lys allele is associated with hypoemotionality [90], ADHD traits [91], mood disorders [92], task switching demands [93] and a reduced capacity to learn from negative feedback [94]. Any presence of the DRD2 Lys (A1) allele decreases dopamine neurotransmission (score = 0); Glu/Glu individuals, score = 1.

The question arose whether polymorphisms that increase DRD2 signaling should be coded in the same direction as those that increase DRD1 signaling given that D1-like receptors activate, and D2-like receptors inhibit, adenylyl cyclase. However, a great deal of evidence suggests that DRD1 and DRD2 act in synergy [95], [96], [97], [98], and that the regulatory balance of dopamine signaling is optimized when these two receptor types work in concert [95]. Consequently, enhanced signaling in D1-like and in D2-like receptors were coded in the same direction.

#### DRD3 (rs6280)

DRD3 is a dopamine receptor that has a SNP resulting in a Ser to Gly substitution at position 9. Dopamine has an affinity to the Gly variant that is 4–5 times higher than its affinity to the Ser variant, and in response to dopamine the Gly variant more robustly increases cAMP inhibition [99]. The Ser^9^Gly DRD3 polymorphism is also associated with an increased risk of tardive dyskinesia, a dopamine supersensitive state [100], and better clinical response to antipsychotic drug treatment [101]. Any presence of the Gly9 DRD3 variant increases dopamine neurotransmission (score = 1); Ser/Ser individuals received a score = 0.

Note that the impact of each polymorphism on dopamine neurotransmission was classified according to its primary effect on brain function; compensatory changes in dopamine, acetylcholine, and other neurotransmitters are not modeled in the current gene score.

### Statistical Analysis

Collection and analysis of MNT and TMS data were performed blind to drug condition and genotype. The main measure of *skilled motor learning* was the improvement in MNT performance, measured as 100-well sequence TTC, over the week. The main measure of *cortical plasticity* was the change in TMS map area, which is known to increase over days of skilled motor training [102]. Map area was calculated as the number of positive sites multiplied by 1 cm^2^.

Statistical analysis was performed using R, specifically the “nlme” package used to build mixed-effects models [103]. To study motor skill learning, we modeled the expected TTC value, *E(TTC)*, as a function of drug condition, gene score, and their interaction. The model controlled for age, gender, ethnicity, and weight (to account for between-subject differences in mg/kg of L-Dopa). In addition, it included day, week, and their interaction in order to model improvement across training days and to account for any differences in difficulty between the week 1 and week 2 MNT boards. Our mixed-effects model includes a subject-specific intercept (i.e., a random intercept) to account for between-subject variation. All other effects were assumed to be fixed. To study cortical plasticity, we used a separate mixed-effects model, where the change in the cortical map area was regressed against drug, gene score, gene score*drug interaction, age, gender, ethnicity, and weight. This model also included a random intercept to account for the paired nature of the two measurements obtained from each participant in week 1 and week 2. Simple linear regression models were used to determine associations of demographic variables with gene score, and Chi squared tests were used to assess Hardy-Weinberg equilibrium. Initial analyses of the gene score weighted all genes equally, but subsequently a partial least squares (PLS) method was used to create a weighted gene score, with the weights for each gene coming from the first component of the PLS analysis of TTC (motor learning) data. Akaike information criterion (AIC) values were calculated to represent the goodness-of-fit for mixed-effects models. To explore single-gene relationships, the single-gene score in Table 1 was inserted into the same mixed-effects model used with the multi-gene score. Significance level for effects was set at α<0.05, with primary hypotheses focused on effects of the gene score. Secondary hypotheses focused on effects of single genes.

## Results

A total of 50 participants (26 M/24F, average age 20.5±2.4) completed the study, demographic details for whom appear in Table 2. Demographic and baseline data had no relationship with the gene score with two exceptions: higher gene score was associated with higher body weight (p = .01) and higher age (p = .05), and so these two measures were included as covariates in subsequent statistical models. The washout period between the L-Dopa week and the placebo week was 15.1±1.5 (mean ± SD) days. There were 5 reports of side effects (nausea, dizziness, or fatigue) on L-Dopa, and 4 on placebo, and the gene scores did not differ between those with (2.2±1.6) versus without (2.2±1.5) side effects. Participants remained blinded to drug condition, as at the end of the L-Dopa week, 60% of participants guessed they had taken placebo, while at the end of the placebo week, 56% of guesses were placebo. The pre-training TMS map area did not differ between the two weeks (p>.1). All five genes were in Hardy-Weinberg equilibrium.

**Table 2:** Demographic and baseline data.

	All	Gene Score						p
		0	1	2	3	4	5	
N	50	5	7	17	13	7	1	
Frequency		10%	14%	34%	26%	14%	2%	
Age	20.5±2.4	19.6±1.1	20.3±1.6	19.9±1.6	20.5±2.1	22.4±4.8	22.0	0.05
Handedness	1.8±0.3	1.8±0.2	1.8±0.3	1.8±0.4	1.9±0.2	1.9±0.1	2.0	0.26
Gender								0.14
Male	26	0	4	9	9	4	0	
Female	24	5	3	8	4	3	1	
Weight (kg)	69.2±12.7	61.9±18.1	59.9±9.6	70.8±10.8	70.8±12.0	76.3±13.7	73.8	0.01
Pill taken first								0.41
L-Dopa	25	2	3	11	8	1	0	
Placebo	25	3	4	6	5	6	1	
Ethnicity								0.12
White	18	0	4	4	3	6	1	
Asian	16	1	2	8	5	0	0	
Hispanic or Latino	9	3	0	3	3	0	0	
African American	1	0	0	0	1	0	0	
Pacific Islander	1	0	0	1	0	0	0	
Other	5	1	1	1	1	1	0	
Baseline time to complete MNT 100-target wells (sec)	259±64	254±50	254±27	254±55	266±90	268±80	257	0.59
Baseline TMS map area (cm^2^)	7.1±3.7	6.8±4.3	7.6±2.2	6.4±3.3	6.0±3.6	10.1±4.7	11.0	0.22
Baseline TMS LMT	50.2±12.9	48.2±11.4	45.9±10.1	51.6±15.9	50.3±11.7	52.4±13.9	51.0	0.45

Results are mean ± SD. The p values are for correlation with the gene score.

**Figure 3:**
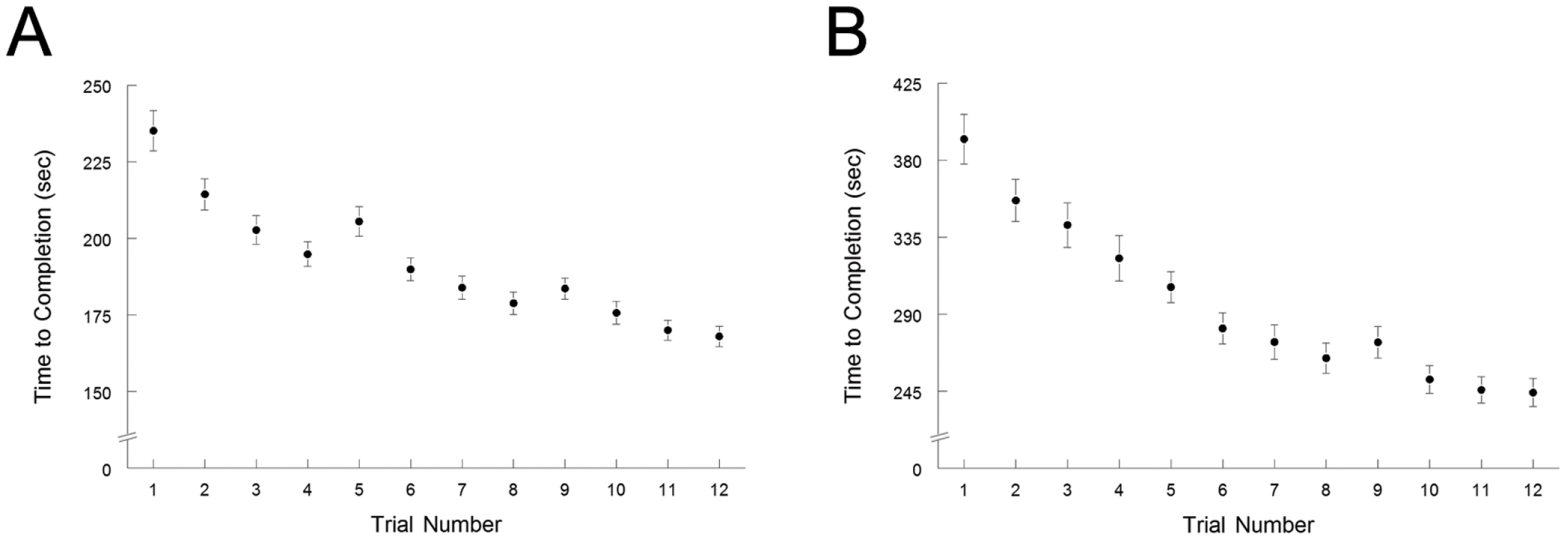
Motor learning data across each week. The average time to completion of each 100-target trial for days 1–3 (4 trials per day) on the MNT for (A) week 1 and (B) week 2. Mean ± SEM.

Significant skilled motor learning on the MNT occurred during both weeks (Figure 3). The slope over each week was negative, indicating a progressive reduction in the time to complete the target sequences (p<.0001). At baseline, MNT TTC was not related to gene score (p = .59).

To examine skilled motor learning, the following mixed-effects model was used:
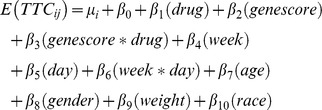
where TTC_ij_ is the jth observation for the ith subject, µ_i_ is the random effect (i.e., subject-specific intercept), and β_0_ to β_10_ are the fixed effects.

Results for motor learning support the three main study hypotheses, and together indicate that lower gene scores are associated with poorer learning on placebo and with a greater boost in learning with addition of L-Dopa (Table 3). Regarding the first hypothesis, the main effect of gene score was significant (estimate for β_2_ = −11.5, p = .03). Thus during the placebo week (value for drug = 0 in the mixed-effects model), higher gene scores were associated with greater skilled motor learning. For every 1 point increase in gene score, the expected TTC declined by 11.5 seconds, indicating better learning. Results also support the second hypothesis, with the main effect of drug being significant (estimate for β_1_ = −11.1, p = .04) and the direction of β_1_ indicating that overall L-Dopa improved learning. However, this result must be interpreted in light of the fact that the gene score*drug interaction term was also significant (estimate for β_3_ = 5.7, p = .008), supporting the third hypothesis. Indeed, the effect of L-Dopa on learning was not significant when genetic information was removed from the model (p = .48). Note that age, gender, weight, and ethnicity (β_7_–β_10_) were not significantly related to learning.

**Table 3:** Results of the mixed-effects model in relation to skilled motor learning.

	Estimate	Std.Error	p-value
**Gene score**	−11.5	5.1	0.03
**Drug**	−11.1	5.5	0.04
Gene score*drug	5.7	2.1	0.0075
**Effect of drug in subset of gene scores 0 and 1**	−24.2	6.01	<.0001
**Effect of drug in subset of gene scores 2–5**	9.4	3.7	0.01

Estimates of the β values for the fixed effects in the model predicting the expected time to completion of the marble game [E(TTC)], with lower times indicating greater motor skill. This model controlled for learning effects across week 1 and 2 by including week, day, and their interaction. The model also controlled for age, gender, weight and ethnicity.

**Figure 4:**
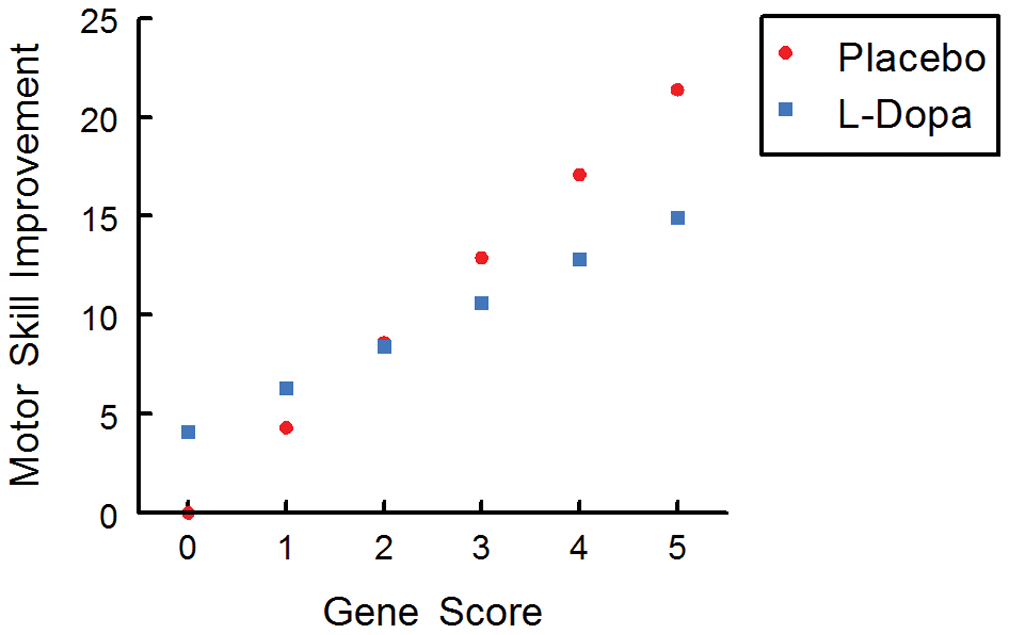
Effect of L-Dopa on skilled motor learning varied with gene score. Below gene score = 2, L-Dopa provides better learning, and above gene score = 2, Placebo provides better learning. Values are derived from the mixed-effects model and reflect the percent improvement from the reference condition of gene score = 0 during the placebo week, using the average value for all covariates.

An additional insight provided by the model is that participants with higher gene scores showed poorer learning with addition of L-Dopa, as compared to placebo (Figure 4). During the L-Dopa week (value for drug = 1 in the mixed-effects model), the expected MNT TTC decreased by 11.1 seconds (main effect of drug), and in parallel TTC increased by 5.7 seconds for every 1-point increase in gene score (gene score*drug interaction term). Once the gene score reaches 2, the beneficial effect of L-Dopa on learning no longer outweighs the interaction term effects, thus L-Dopa no longer provides a benefit for participants with gene scores of 2 or greater. Post-hoc analyses were consistent, suggesting that the gene score improved the ability to predict which participants would benefit from L-Dopa: in the subset of participants with lowest gene scores (0 or 1 only, 24% of participants), learning was significantly greater in the L-Dopa condition (estimate of β = −24.2, p<.0001), but in the subset with all other gene scores (score 2–5 only, 76%) L-Dopa had a detrimental effect (estimate of β = 9.4, p = .01). Additional post-hoc analyses were performed to determine whether the differential difficulty of the week 1 and week 2 MNT boards affected results, by adding gene*week and drug*week interactions to the model; 3-way interactions were not considered to avoid over-parameterization of the model. The drug*week interaction was not significant (p = 0.49), but the gene*week interaction was (estimate of β = −8.9, p = 0.0001), indicating that L-dopa did not have a differential effect between the 2 weeks, but gene score did, with higher gene scores associated with greater learning during week 2 relative to week 1.

Regarding cortical map plasticity, the gene score*drug interaction term was significant (estimate of β = 1.5, p = .02): during the placebo week (change in map area 0.4±3.5 cm^2^), participants with lower gene scores showed greater map enlargement, while during the L-Dopa week (change in map area 0.8±3.8 cm^2^), participants with higher gene scores showed greater map enlargement. These findings do not simply reflect a change in cortical excitability, as participants’ lowest motor threshold (LMT) did not change significantly across the four TMS sessions, and TMS map size did not correlate with LMT at any session. Regarding the potential impact of differential difficulty of the week 1 and week 2 MNT boards, a paired t-test found no significant difference in map expansion between weeks 1 and 2 (p = 0.35). Post-hoc testing found that the gene*week and the drug*week interaction terms were not significant when added to the model; again, a 3-way interaction was not considered, to avoid over-parameterization of the model. The main effects of drug and of gene score were not significant, and the degree of map expansion was not significantly correlated with degree of motor learning.

The above analyses assumed equal weighting of each polymorphism, but the five polymorphisms might influence learning and plasticity to differing extents. This was examined by creating a weighted gene score. The weighted score demonstrated substantial improvement over the unweighted score, indicated by lower AIC values (Table 4). Note that although the weights were determined using the skilled motor learning data, the weighted score also improved the model fit when applied to the TMS cortical plasticity data.

**Table 4:** Gene scores in relation to learning and plasticity.

Score	Motor Learning		TMS map plasticity	
	AIC	p	AIC	p
Weighted 5-Gene score	12537.2	0.002	557.8	0.89
Unweighted 5-Gene score	12632.2	0.008	559.9	0.02
Gene score WITHOUT DRD1	12632.6	0.01	560.1	0.02
Gene score WITHOUT DRD2	12639.8	0.26	561.0	0.03
Gene score WITHOUT DRD3	12634.0	0.02	560.6	0.04
Gene score WITHOUT DAT	12633.1	0.007	562.9	0.08
Gene score WITHOUT COMT	12626.1	0.0003	558.1	0.007
DRD1 only	12641.3	0.3	565.5	0.3
DRD2 only	12621.2	<.0001	561.7	0.3
DRD3 only	12638.2	0.06	561.4	0.07
DAT only	12640.5	0.4	561.9	0.04
COMT only	12641.4	0.34	566.3	0.9

AIC is a measure of goodness of fit, where lower numbers indicate a better fit. P-values are for the gene score*drug interaction term. The unweighted gene score assumed equal contribution from each polymorphism, while the weighted gene score used weights for each polymorphism that were derived from the motor learning task.

While the gene score was more robust than any single polymorphism, some single polymorphisms did show an association with learning or with plasticity. The most significant of these single polymorphism associations was found with the DRD2 polymorphism. Entering the DRD2 polymorphism into the model as the sole source of genetic data showed a significant association with motor learning (estimate for β = −28.3, p = .02) and with the gene*drug interaction for motor learning (estimate for β = 23.4, p<.0001), but not with any cortical plasticity measure, including the gene*drug interaction. Further, the β estimate for each individual gene predicting E(TTC) was in the expected direction (DRD1: β = −5.9, p = .65; DRD3: β = −16.7, p = .25; COMT: β = −0.2, p = .99; DAT: β = −14.3, p = 0.22). Entering the DAT polymorphism only into the models produced a significant association with the gene*drug interaction for cortical plasticity only (estimate for β = 3.2, p = .04). The DRD3 polymorphism predicted TMS map area (estimate for β = −2.9, p = .04). Note, however, that the significance of these single polymorphism associations is less clear if one formally corrects for multiple comparisons. Such single gene results show that several polymorphisms are contributing to the gene score results. Data regarding the DRD1 and COMT polymorphisms alone did not show any associations with the motor learning or plasticity endpoints. Thus, no individual gene accounted for the full constellation of findings associated with the gene score. Consistent with this, use of a 4-gene score, i.e., successively leaving out one SNP at a time, resulted in weakened but not altered relationships in almost all cases (Table 4). The sole exception was the 4-gene score omitting COMT, which resulted in a better fit for the model than with the full gene score. Thus the dopamine gene score benefits from the contributions of all five initially hypothesized polymorphisms, with the possible exception of COMT.

Reanalysis of results using an allele score, where each gene had a score of 0, 1, or 2 based on the number of polymorphic alleles, showed very similar results to above.

## Discussion

The current study found that a gene score reflecting the collective influence of five dopamine-related polymorphisms is associated with a significant difference in skilled motor learning, and with a significant difference in the effect that L-Dopa has on motor learning and motor cortex plasticity. Dopamine genotype was not associated with differences at baseline, but instead through interaction with experience, similar to prior genetics studies [104]. Importantly, the gene score significantly improved the ability to predict participants whose motor learning benefited from L-Dopa (score of 0–1) as opposed to those where learning was impeded by the drug (score of 2–5). The results suggest that a gene score is useful for capturing the effects of genetic variation on dopamine neurotransmission, and that such data might be useful to more precisely prescribe dopaminergic therapies.

The current approach examined dopamine neurotransmission collectively. Thus while dopamine is involved in many different neurobiological events across several brain regions, the model underlying the current approach is that genetic influences on dopamine neurotransmission in the motor system are additive. This model builds on observations in the experimental literature, where dopamine levels have been found to be related to motor learning and plasticity in a dose-dependent manner, with increased dopamine promoting motor learning and motor cortex plasticity [51], [105], and decreased dopamine impairing motor learning [106] and motor cortex plasticity [105]. Significant evidence exists that enhancements associated with increased dopamine occur in an inverted U-shaped manner [7], [107], [108], [109]. That is, an excess of dopamine can be deleterious, resulting in dyskinesia [110], [111], impaired working memory [7], impulsive-antisocial traits [112], and impulse control disorders such as pathological gambling [113], [114]. The current results are congruent with this inverted U-shaped curve. On the one end of this curve, learning improved when L-dopa was given to individuals with low gene scores, i.e., with low endogenous dopamine neurotransmission; these subjects were moved towards the optimal point. However, learning was impeded when L-Dopa was given to individuals with high gene scores, i.e., with high endogenous dopamine neurotransmission; these subjects are higher on the curve at baseline, addition of L-dopa moves them past the optimal point, and learning suffers. The pattern of the inverted U-shaped curve was also suggested by L-Dopa effects on TMS map area, with subjects at the two extremes of the curves (low dopamine, i.e., low gene scores on placebo, and high dopamine i.e., high gene scores on L-dopa) showing the largest degree of map expansion.

This research also points to a significant involvement of the DRD2/ANKK1 (rs1800497) polymorphism in motor learning and its modulation by L-Dopa. With the Lys allele present and thus DRD2 dopaminergic tone reduced, the expected TTC on the motor learning task in the placebo condition is worsened by 28.3 seconds, and this effect is virtually eliminated in the L-Dopa condition; no associations were found between the DRD2 polymorphism and cortical plasticity measures. These data suggest that the DRD2 polymorphism is an important modifier of learning effects and their modulation by L-Dopa, although note that the maximum effect of variation in DRD2 on MNT TTC (28.3 seconds) is much smaller than the maximum effect of variation in the gene score (57.5 seconds). Further, examining the two predictors emphasizes the greater potential utility of the gene score. Consistent with the underlying motivation for principal component analysis (PCA), higher variance indicates an increased potential for explaining the variability of response variables without pre-specification of any one response variable. In our data, the sample variance for the gene score is 1.5, whereas the sample variance of DRD2 was only 0.41. Thus while the DRD2 polymorphism had good explanatory value for one specific response variable (i.e., motor learning), it has a lower sample variance compared to the gene score and so is less robust for capturing the heterogeneity of individuals across a wider range of possible response variables. The gene score serves as a more robust predictor across a variety of dopamine-related endpoints; the DRD2 polymorphism, although having significant associations with altered dopaminergic neurotransmission, benefits from the addition of other dopamine genetic polymorphism data when examining a broad spectrum of behaviors.

It is possible that improved learning occurred due to enhanced reward, motivation, or attention to task. L-dopa has been shown to bias reward prediction [115], so conceivably L-dopa effects were in part related to enhanced reward resulting from MNT performance improvements. In support of this, a 5-gene score similar to that used herein was found in a prior study to be associated with fMRI signal change in response to reward, within reward-related brain areas. Specifically, participants with gene scores associated with lower dopamine neurotransmission had lower fMRI BOLD responses to reward [116]. As such, modified reward bias might have influenced motor skill acquisition in the current study.

There is evidence that more difficult tasks activate the endogenous dopamine system to a greater extent [117]. Consistent with this, the week*gene score (week being a proxy for task difficulty given use of the more difficult task in week 2) interaction term for learning was significant, with higher gene scores associated with greater learning with the more difficult task of week 2, relative to week 1. It is unclear why subjects with higher gene scores showed improved learning when dopamine increased as a result of a more difficult task, but poorer learning when dopamine was increased as a result of L-Dopa administration. This finding suggests that the endogenous boost in dopamine resulting from increased task difficulty is smaller than the boost provided exogenously by 100 mg of L-dopa, and so does not move individuals with higher gene scores past the optimal point on the inverted U-shaped curve; such a difference between an endogenous vs. exogenous dopamine boost might also explain why subjects with lower gene scores did not show improved learning in week 2. Alternatively, there may simply be some differences in the precise pathways activated by task-related vs. exogenous dopamine.

The use of a gene score might be criticized because dopamine is involved in many different events distributed across many brain regions, and so has the disadvantage that multiple neural processes are examined in combination. However, the effect of L-Dopa on behavior is an aggregate reflection of changes in dopaminergic neurotransmission at several brain areas, which is mediated by multiple dopamine-related proteins. The overall approach of the current investigation is to measure the net effect of these multiple dopaminergic events in the brain. In support of this approach, the goodness of fit (AIC, Table 4) for the gene score surpassed that of any individual polymorphism for identifying biologically distinct subgroups with respect to motor learning, plasticity, and drug response. The findings therefore support the utility of a combined approach to studying genetic influences on a system such as dopamine neurotransmission.

The gene score predicted the effect of L-Dopa on learning, as participants with lower gene scores, who therefore had lower dopamine neurotransmission, showed poorer learning on placebo and a greater boost in learning from L-Dopa, as compared to participants with higher gene scores. These polygenic results echo prior studies that were focused on a single polymorphism, COMT val^158^met. These studies found that participants lacking the polymorphism, and who therefore have reduced dopamine neurotransmission, had a greater boost in cognitive performance with addition of amphetamine, as compared to participants possessing this polymorphism [7], [66]. Together, these results support the interpretation that genetic variation can be associated with reduced dopamine neurotransmission, and furthermore suggest that exogenous medication can overcome this genotype-associated trait.

The gene score also predicted the effects of L-Dopa on motor cortex plasticity. Among participants with lower gene scores, plasticity was greater during the placebo week, while among participants with higher gene scores, plasticity was greater during the L-Dopa week. Previous studies have shown that map expansion occurs with short-term learning and plasticity, and that TMS map expansion is generally regarded a sign of adaptive cortical plasticity over days of motor learning [118], [119], [120]. One interpretation of the current results is that the further individuals were from the optimal point on the inverted U-shaped curve, the more motor cortex plasticity was invoked. Individuals with lower gene scores were furthest from the optimal point during the placebo week, and individuals with higher gene scores were furthest from the optimal point during the L-Dopa week, and these are also the situations when TMS map expansion was maximal. A recent fMRI study in healthy young participants by Krebs et al [117] potentially provides insights into the basis for the effect of the gene score*drug term in relation to current motor cortex plasticity findings. These authors found that cues associated with greater task difficulty were associated with increased activation in a circuit that included the dopaminergic midbrain (ventral tegmental area) and cerebral cortex (such as medial frontal and dorsolateral prefrontal cortex). Dopaminergic activity increases when the demand level of a task is greater. This is consistent with models of dopaminergic activity in relation to behavioral status with Parkinson’s disease [109], and might explain the greater degree of cortical map expansion when subjects in the current study were furthest from the optimal point of the dopamine inverted U-shaped curve. This overall pattern–increased cortical recruitment when the brain falls from an optimal state, such as with greater task difficulty–has been described in other settings as well, for example, during movement performance in the setting of multiple sclerosis [121], Huntington’s disease [122], or normal aging [123], [124]; and during working memory task performance in the setting of schizophrenia [3]. Whether increased cortical recruitment in these diverse contexts is driven by a fall from the optimal state in the dopamine system, or by events in other systems, remains to be clarified.

The five dopamine-related polymorphisms predicted skilled motor learning, and the effect that L-Dopa has on motor learning and motor cortex plasticity, better when combined as a gene score than when examined alone. The weighted gene score consistently showed the strongest correlations with learning and plasticity, with AIC values lower than those of any single gene or leave-one-out score. Because these weights were derived from a motor learning paradigm in healthy young individuals, it is possible that different weights would be needed when studying different behavioral measures or different populations. A gene score has previously been found useful for measuring the combined effects of multiple polymorphisms in a number of biological settings, such as breast cancer [23], prostate cancer [24], coronary artery disease [25], and the dopamine reward system [125]. Possibly, an expanded dopamine gene score that considers additional dopamine-related polymorphisms will demonstrate a greater degree of explanatory power.

The current study has several strengths and limitations. Learning, genetic, pharmacological, and neurophysiological measures were studied in parallel, providing a broad spectrum of perspectives to interpret the findings. Also, in order to focus on the dopamine gene score, individuals carrying the Met allele for the BDNF val^66^met polymorphism were *not* included in the study. The BDNF val66met SNP has significant effects on several forms of motor cortex plasticity and motor learning [12], [44], [126], [127], and so we controlled for the presence of this polymorphism by screening and excluding any BDNF val^66^met polymorphism carriers from the current study; all participants were val/val. Controlling for the BDNF val^66^met SNP in this way allowed analyses and interpretations to focus on dopamine genetics; however, uncertainty remains as to whether the current dopamine-related findings would differ among individuals who do carry the BDNF Met allele. The current study was not adequately powered to examine interactions between the dopamine polymorphisms, although few such interactions have been reproducibly found in the literature, and the leave-one-out approach (Table 4) generally weakened genetic associations with learning and plasticity. Only a single dose level of L-Dopa was examined, and associations with the gene score might differ at higher drug doses. Some genetic polymorphisms have uncertain associations with dopaminergic signaling, and negative studies have been reported for each of the polymorphisms used in the current gene score [128], [129], [130], [131]. Dopamine effects are influenced by numerous factors such as the dynamics and concentration of its release [10], [132], issues not examined in the current study. Bilder et al hypothesize that the effects of the COMT polymorphism differ between the cortex and striatum and between tonic vs. phasic dopamine release. Given that motor learning affects both cortical [43], [133] and striatal [40] parameters, confounds between COMT effects on cortical vs. striatal dopaminergic function may partially explain why this polymorphism did not benefit our unweighted gene score. Finally, task order was not randomized in the current study.

The current results could be of substantial clinical significance if confirmed in patient populations. Dopaminergic drugs have long been studied towards the goal of improving outcome via enhanced brain plasticity after neural injury such as stroke. However, results to date have been inconsistent. The current findings in healthy young adults might inform this issue, as a dopamine agonist provided benefit to the 24% of the population with a gene score of 0 or 1, but was detrimental to 76% of participants with higher scores, possibly corresponding to the inverted U-shaped dopamine dose-response curve found in other studies [7]. Furthermore, modulating brain dopaminergic neurotransmission is the therapeutic focus for many other conditions such as Parkinson’s disease, where therapeutic response to dopaminergic therapy varies widely across patients [14]. For dopaminergic therapies, improved methods are needed to select the best prescription for an individual patient, guide therapeutic decision making [134], and stratify patients in clinical trials [135]. The current study addressed this issue by studying a gene score that examined multiple genetic influences collectively, and this approach provided significant improvements in predicting motor learning. The results suggest that a gene score is useful for capturing the effects of genetic variation on dopamine neurotransmission, and that such an approach may be useful for understanding inter-individual differences in motor learning, plasticity, and the response to a dopaminergic drug.

## Supporting Information

Methods S1 (DOC)
