# Supplementary material for: Genetic Variation in the Human Brain Dopamine System Influences Motor Learning and Its Modulation by L-Dopa
Source: PLoS One. 2013 Apr 17;8(4):e61197. doi: 10.1371/journal.pone.0061197 (PMC3629211; doi:10.1371/journal.pone.0061197)
Supplement: Methods S1 — (DOC) [file pone.0061197.s001.doc]

**Supplemental Methods**

*Genotyping*

DNA extraction--DNA for genotyping was extracted from whole blood by salt precipitation. Genotyping for all polymorphisms was performed using polymerase chain reaction (PCR) - restriction fragment length polymorphism analysis. PCR products were digested with the appropriate restriction enzymes, digestion products were run on agarose gel and bands were visualized with ethidium bromide. Choice of primer sequences and digestion enzymes followed established protocols, as indicated below.

Catechol-O-methyltransferase (COMT)--For the Val^158^Met amino acid substitution on the COMT gene (rs4680), the COMT PCR product was digested with NlaIII .

Dopamine active transporter (DAT)--This study examined a 40 base pair variable number of tandem repeats (VNTR) on the DAT gene (rs28363170). The DAT gene was amplified using (DATVNTRF: 59-TGTGGTGTAGGGAACGGCCTGAG-39 and DATVNTRR: 59-CTTCCTGGAGGTCACGGCTCAAGG-39) .

DRD1--For the -48 A to G SNP on the DRD1 gene (rs4532), the DRD1 PCR product was digested with DdeI .

DRD2--For the Glu^713^Lys amino acid substitution (Taq1A polymorphism, rs1800497) on the gene for ANKK1, situated close to DRD2 and associated with DRD2 density, amplified PCR products were digested with the TaqI restriction enzyme .

DRD3--For the Ser^9^Gly amino acid substitution in the DRD3 gene (rs6280), DRD3 PCR products were digested with MscI .

BDNF--For the BDNF val^66^met polymorphism (rs6265), evaluated as part of the study screening process, amplified PCR products were digested with the restriction enzyme Hsp92II, and also with EcoR1 to confirm the results .

*References for Supplemental Methods*

1. Berthele A, Platzer S, Jochim B, Boecker H, Buettner A, et al. (2005) COMT Val108/158Met genotype affects the mu-opioid receptor system in the human brain: evidence from ligand-binding, G-protein activation and preproenkephalin mRNA expression. Neuroimage 28: 185-193.

2. Kang AM, Palmatier MA, Kidd KK (1999) Global variation of a 40-bp VNTR in the 3'-untranslated region of the dopamine transporter gene (SLC6A3). Biol Psychiatry 46: 151-160.

3. Limosin F, Loze JY, Rouillon F, Ades J, Gorwood P (2003) Association between dopamine receptor D1 gene DdeI polymorphism and sensation seeking in alcohol-dependent men. Alcohol Clin Exp Res 27: 1226-1228.

4. Noble EP, St Jeor ST, Ritchie T, Syndulko K, St Jeor SC, et al. (1994) D2 dopamine receptor gene and cigarette smoking: a reward gene? Med Hypotheses 42: 257-260.

5. Woo SI, Kim JW, Rha E, Han SH, Hahn KH, et al. (2002) Association of the Ser9Gly polymorphism in the dopamine D3 receptor gene with tardive dyskinesia in Korean schizophrenics. Psychiatry Clin Neurosci 56: 469-474.

6. Cheeran B, Cohen L, Dobkin B, Ford G, Greenwood R, et al. (2009) The future of restorative neurosciences in stroke: driving the translational research pipeline from basic science to rehabilitation of people after stroke. Neurorehabil Neural Repair 23: 97-107.
